# Effects of Parasitism on the Competitive Ability of Invasive and Native Species

**DOI:** 10.3390/life12111800

**Published:** 2022-11-06

**Authors:** Yongge Yuan, Junmin Li

**Affiliations:** 1 School of Advanced Study, Taizhou University, Taizhou 318000, China; 2 Zhejiang Provincial Key Laboratory of Plant Evolutionary Ecology and Conservation, Taizhou University, Taizhou 318000, China

**Keywords:** competition, *Cuscuta grovonii*, native plant, parasitic plant, invasive plant

## Abstract

Parasitic plants can often seriously harm host plants and, thus, alter competitive dominance between hosts and neighbouring species. However, whether and how parasitic plants differently affect the competitive abilities of invasive and the native plants have not been tested. In this study, we used *Cuscuta grovonii* as the parasitic plants and three invasive plants and three native plants as host plants. Host plants grown alone or in competition with *Coix lacryma-jobi* were either parasitized with *Cuscuta grovonii* or not parasitized. Parasitism caused similar damage to invasive and native plants when grown with *Cuscuta grovonii* alone but caused less damage to invasive species than native species when grown in competition. Parasitism increased the competitive ability of invasive plants but did not affect the competitive ability of native plants. In the absence of parasitism, the competitive ability of host plants was significantly negatively correlated with the competitive ability of *Coix lacryma-jobi*, but under parasitism, there was no significant relationship of the competitive ability between host and competitor plants. Our results indicated that parasitic plants can increase the competitive tolerance of invasive plants, but have no effect on native plants. Thus, parasitism may play an important role in the process of plant invasion.

## 1. Introduction

Plant invasion has caused serious economic losses and threatened the biodiversity of ecosystems [1,2] and studies on the mechanisms underlying the success of plant invasions have been an active research focus in ecology and environmental sciences. After arriving in a new habitat, alien plants re-establish new interactions with multitrophic organisms, which may have the potential to facilitate or hinder their invasion [3]. For example, herbivory can promote, inhibit, or have no effect on plant invasion [4,5,6,7]. Therefore, exploring the interactions between invasive species and their new interactors as well as their consequent ecological effects are of great importance to find effective ways to manage invasive plants.

Invasive plants often differ from native plants in their competitive ability [8,9,10], but their effects on native plants can be inconsistent. Some invasive plants are considered to be more competitive than native plants because of resource reallocation from defence to growth or competition (as characterized by the evolution of increased competitive ability (EICA) hypothesis) [11], strong allelopathy [12], or enhanced mutualism [13,14]. However, other studies showed that not all invasive plants are more competitive than native plants and the competitive ability of invasive plants can vary with competitors [15], herbivore stress, or local environments [16]. For example, Sakata and Craig [16] showed that herbivores differently affected the competitive abilities of invasive and native plants and reinforced the negative effects of exotic plants on native plants in herbivore-dense environments. Therefore, the biological control of invasive plants can be advanced by finding biotic factors that can shift the competitive hierarchy between native and invasive plants.

Parasitic plants are heterotrophs that obtain part or all of their water, carbon, and nutrients from host plants via haustoria [17,18,19]. Parasitic plants can suppress the growth of invasive plants and have been considered effective biocontrol agents [18]. Parasitic plants often cause serious damage to the growth of host plants and, thus, alter the competitive dominance among host plants and neighbouring species [20,21]. For example, Li et al. [18] found that parasitism on the host species *Mikania micrantha* by the parasitic plant *Cuscuta campestris* decreased the growth of *M. micrantha* and facilitated the growth of neighbouring *Coix lacryma-jobi* plants. However, whether and how parasitic plants differently affect the competitive abilities of invasive plants and native plants have not been tested.

Invasive plants may respond to parasitism differently from native plants. On one hand, parasitic plants may have a larger impact on invasive species than native species, because invasive species generally have higher resource use efficiency and higher nutrient content than native species [22,23,24]; thus, parasites can acquire more resources from invasive plants than those from native plants [24]. On the other hand, invasive plants may have evolved lower resistance to parasitism than native plants in accordance with the EICA hypothesis [25,26]. In this study, we used *Cuscuta grovonii* as the parasitic plant to evaluate the competitive abilities of three invasive plants and three native plants in the absence or presence of parasitic plants. *Cuscuta grovonii* was chosen because it is holoparasite, which is common in the southeast of China [27]. We observed that *C. grovonii* can cause serious damage on some invasive and native plants in the field, including *Solidago canadensis*, *Bidens pilosa*, and *Lepidium virginicum*. Our objective was to test how the parasitic plant differently regulates the competition between invasive plants and native plants. Our hypotheses were as follows: (1) parasitic plants would cause more damage to invasive species than native species; (2) parasitic plants would decrease the competitive ability of invasive plants more than that of native plants. Our results not only enrich the understanding of the ecology of invasive plants but also provide a useful guide for the management of invasive plants.

## 2. Materials and Methods

### 2.1. Plant Species and Cultivation Conditions

We selected three invasive plant and three native plant species pairs belonging to three different families (Compositae, Amaranthaceae, Cruciferae, respectively) as host plants. The three invasive plants were *Solidago canadensis*, *Alternanthera philoxeroides*, and *Lepidium virginicum*. The three native plants were *Ixeris polycephala*, *Achyranthes bidentata*, and *Cardamine hirsuta*. The selection of pairs of invasive and native plant species in the same family was intended to control for phylogenetic effects. We also selected one native plant species *Coix lacryma-jobi* (hereinafter *Coix*) belonging to Poaceae as the native competitor in our study. The stems of *C. grovonii* were collected from a field population near Taizhou University, Taizhou, China.

The three paired invasive and native plants and *Coix* are common host plant species in field which can be parasitized by *C. grovonii*. *Solidago canadensis* is native to North America and was first introduced to China in the 1910s. It then rapidly spread along the southeast coast of China [28]. *Alternanthera philoxeroides* is native to South America and was widely introduced to Southern China [29]. *Lepidium virginicum* is native to North America and now mainly distributed in the east of China [30]. *Ixeris polycephala*, *A. bidentata*, *C. hirsute*, and *Coix* are common native plant species that are widely distributed in China (http://www.iplant.cn/frps, accessed on 3 November 2022). It is highly likely that these six host plants can compete with *Coix* in field.

Three-centimetre-long stem cuttings of *Alternanthera philoxeroides* were collected from a field near Taizhou University. The stem cuttings of *A. philoxeroides* were vertically inserted into a plastic box (60 cm × 30 cm × 20 cm) filled with peat and cultivated in a greenhouse. Seeds of the other six species were bought from a commercial supplier.

On 20 June 2018, seeds of the plant species, except *A. philoxeroides*, were surface sterilized for 10 min in 5% sodium hypochlorite. After that, the sterilized seeds germinated in a sterilized mixture of vermiculite and peat moss (1:1, *v*:*v*).

### 2.2. Experiment Set-Up

The substrate used in this study was a mixture of peat, sand, and vermiculite (6:3:1, *v*:*v*:*v*). The peat had a pH of 6.2 and contained 635 g kg^−1^ organic matter, 8.43 g kg^−1^ total nitrogen, and 0.604 g kg^−1^ total phosphorus. On 31 July 2018, all germinated seedlings were transplanted into plastic pots (diameter × height, 17.5 cm × 14.6 cm) filled with substrate. Additionally, one-node stem cuttings of *A. philoxeroides* measuring 3.0 cm in length and with similar stem diameters were also planted vertically into the substrate in plastic pots.

This experiment had three factors with six replicates for each treatment: (1) six host plant species (three invasive and three native species); (2) with or without parasitism (P+ and P−); (3) two competition statuses (each of the six host species alone, with just one host seedling per pot, or in competition with one *Coix* seedling). Additionally, to test the competitive ability of *Coix*, one extra treatment that consisted of one *Coix* seedling cultured alone in one pot was also performed.

Four weeks after transplantation, one 10 cm long stem piece of *C. grovonii* was wound counter clockwise around the stems of host plants to allow the parasite to infect the host plants. Non-parasitized host plants were treated as controls [31]. The experiment was conducted in a greenhouse with 16 h of light and 8 h of dark at 22 °C (day) and 18 °C (night), respectively. Water was sprayed evenly onto the soil of each container every day.

### 2.3. Harvest

On 30 September 2018, the *C. grovonii* plants were harvested by detaching them from the host plants. After that, shoots of plants were cut 2 cm above the soil surface [32]. Aboveground biomass was dried at 60 °C to a constant mass (at least 72 h) before weighing.

### 2.4. Statistical Analysis

The relative competition intensity (RCI) index was assessed as the difference between biomass when host plants were grown alone and mean biomass when they were grown in competition divided by biomass when grown alone: (Biomass_alone_ − Biomass_competition_)/Biomass_alone_ [9]. To test the effect of invasive status of host plants (i.e., invasive vs. native) and parasitism (P+ vs. P−) on RCI of host plants or on RCI of *Coix*, we used linear mixed-effects models with host invasive status and parasitism as fixed factors and species as a random effect nested in host invasive status after the homogeneity test.

The deleterious effect (DE) index is defined as the difference between biomass without parasitism and mean biomass under parasitism divided by biomass without parasitism: (Biomass_parasitized_ − Biomassun_parasitized_)/Biomassun_parasitized_. To test the effect of the invasive status of host plants (invasive vs. native) and competition status on the DE of *C. grovonii* on host plants, we also used linear mixed-effect models with host invasive status and competition status as fixed factors and species as a random effect nested in host invasive status after the homogeneity test.

Data were transformed, as necessary, using the appropriate transformations. Specifically, these were sine transformation for the RCI index, cube root transformation for the DE index, and natural logarithm (ln) transformation for *C. grovonii* biomass and for the ratio of *C. grovonii* biomass to host biomass.

Spearman’s correlation analysis was used to assess the relationship between the RCI of host plants and the RCI of *Coix* in the absence or presence of parasitism. Additionally, a simple linear regression model (y = a + bx) was used to fit a straight line to the relationship between the DE index on invasive or native plants and the RCI index of invasive or native plants. To determine whether the slopes of the regression lines significantly differed between invasive and native plants, the homogeneity of the slopes (i.e., parallelism) was tested by one-way ANCOVA [33]. SPSS (version 16.0; SPSS Inc., Chicago, IL, USA) was used for all statistical analyses and significance was assessed at a *p* < 0.05 threshold.

## 3. Results

### 3.1. Competitive Ability of Host Plants and Coix

**Figure 1:**
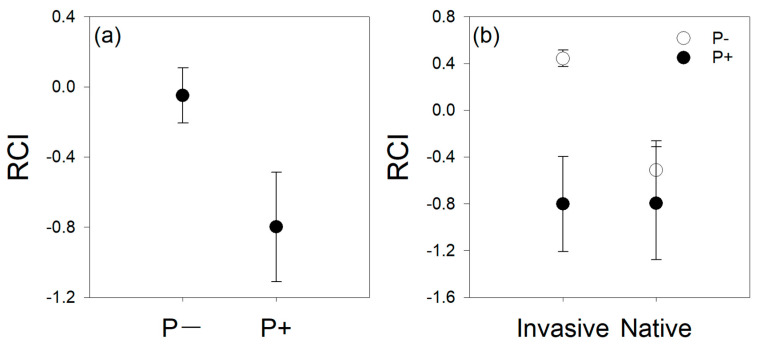
Effect of parasitism on relative competition intensity (RCI) for host plants. (**a**) The main effect of parasitism. (**b**) The main effect of parasitism and host invasive status. RCI was calculated as RCI = (Biomass_alone_ − Biomass_competition_)/Biomass_alone_. P−, without *Cuscuta gronovii* parasitism; P+, with *C*. *gronovii* parasitism.

Parasitism significantly decreased the RCI of host plants (Table 1; Figure 1a). There was a significant interactive effect between parasitism and host invasive status. Specifically, parasitism significantly decreased the RCI of invasive plants, but did not significantly affect the RCI of native plants (Table 1; Figure 1b). In the absence of parasitism, the RCI of invasive plants was significantly higher than the RCI of native plants. However, in the presence of parasitism, there was no significant difference in RCI between invasive and native plants (Table 1; Figure 1b).

**Figure 2:**
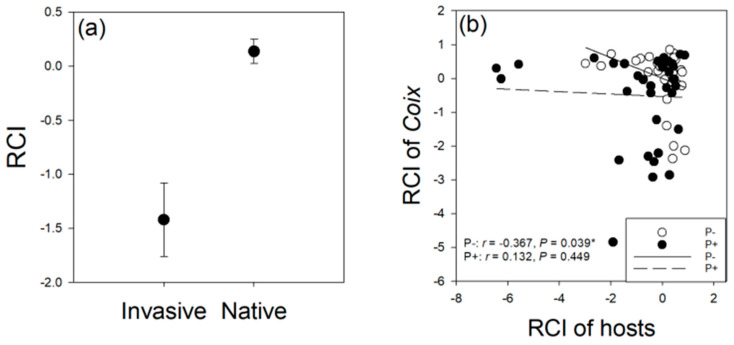
Effect of parasitism on relative competition intensity (RCI) of *Coix lacryma-jobi*. (**a**) The main effect of invasive status of host plants. (**b**) The relationship of RCI between hosts and *Coix*. RCI was calculated as RCI = (Biomass_alone_ − Biomass_competition_)/Biomass_alone_. P−, without *Cuscuta gronovii* parasitism; P+, with *C*. *gronovii* parasitism.

The RCI of *Coix* was significantly lower when in competition with invasive plants than native plants. Parasitism of host plants did not significantly affect the RCI of *Coix* competing with those host plants (Table 1; Figure 2a). In the absence of parasitism, there was a significantly negative relationship in RCI between host plants and *Coix*. Under parasitism, there was no significant relationship of RCI between host plants and *Coix* (Figure 2b).

### 3.2. Deleterious Effect of Cuscuta grovonii on Host Plants

**Figure 3:**
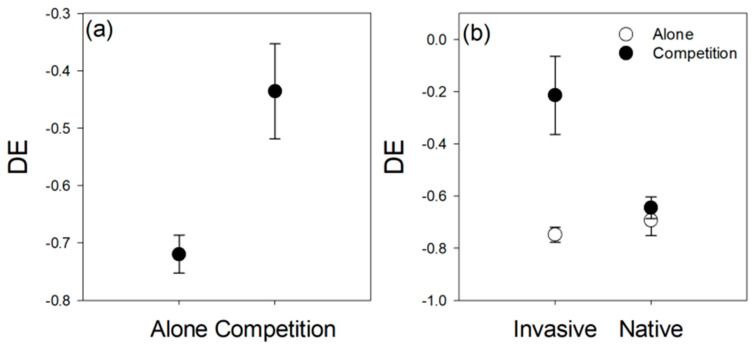
The effect of competition on the deleterious effect (DE) of *Cuscuta grovonii* on host plants. (**a**) The main effect of competition with *Coix lacryma-jobi*. (**b**) The main effect of competition with *Coix lacryma-jobi* and invasive status of host plants. DE was calculated as DE = (Biomass_parasitized_ − Biomass_unparasitized_)/Biomass_unparasitized_.

Competition significantly increased the DE of *C. grovonii* on host plants (Table 2; Figure 3a). There was a significant interactive effect between competition and invasive status on the DE of *C. grovonii* on host plants. Competition significantly increased the DE of *C. grovonii* on invasive plants but did not significantly affect the DE of *C. grovonii* on native plants (Figure 3b). When grown without competition, the damage caused by *C. grovonii* to invasive and native plants was not significantly different, but when grown in competition, *C. grovonii* caused significantly more damage to invasive plants than native plants (Figure 3b).

**Figure 4:**
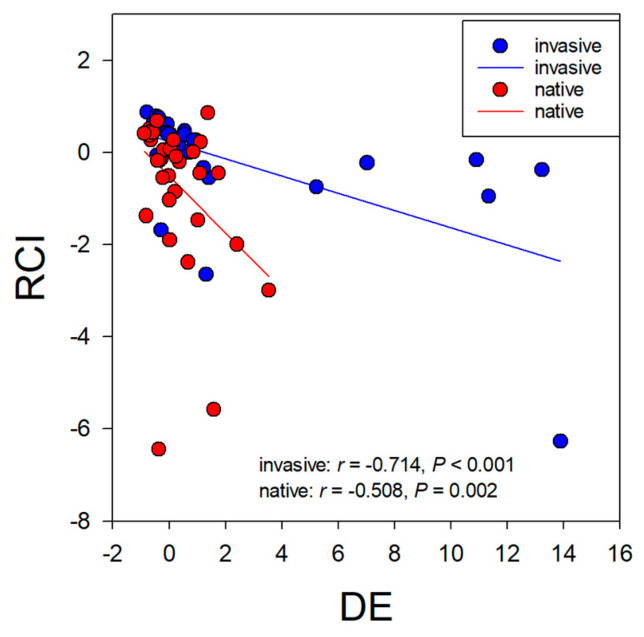
The relationship between relative competition intensity (RCI) of invasive plants and the deleterious effect (DE) of *Cuscuta grovonii* on invasive plants (blue dots and the solid blue line) and native plants (red dots and the solid red line).

The DE of *C. grovonii* on invasive or native plants was significantly negatively correlated with the RCI of invasive or native plants. The slopes of the regression lines were significantly different between invasive and native plants (Figure 4).

### 3.3. The Biomass Ratio of Cuscuta grovonii to Host Plants

Neither competition nor host invasive status significantly affected the biomass of *C. grovonii* or the ratio of *C. grovonii* biomass to the biomass of host plants (Table 3). The ratio of *C. grovonii* biomass to host biomass varied among host plant species. The ratio of *C. grovonii* biomass was highest when parasitizing *Achyranthes bidentata* and *Ixeris polycephala* and was lowest when parasitizing *Lepidium virginicum* (Table 3; Figure S2).

## 4. Discussion

We found that *C. grovonii* parasitism differently affected the competitive abilities of invasive and native plants. Specifically, parasitism increased the effects of competition of invasive plants with *Coix* but did not affect the effects of competition of native plants with *Coix*. Our results were consistent with a previous study, showing that higher-trophic-level organisms (e.g., herbivores or parasites) can regulate the competition between invasive and native plant species [5]. The modulation of the competition of invasive and native plants caused by parasitism might influence the consequent process of plant invasion.

Non-native plants can use two strategies to gain a competitive advantage over native plants, neighbour tolerance and neighbour suppression [34,35]. Neighbour tolerance refers to the ability to tolerate unfavourable conditions associated with the presence of neighbours [34,36], while neighbour suppression refers to the ability to suppress neighbouring plants via superior resource acquisition and/or allelopathic activity [34,35,37]. In this study, we found that in the absence of parasitism, the competitive ability of host plants was significantly negatively correlated with the competitive ability of Coix, indicating that neighbour suppression may be the main competitive strategy between Coix and host plants in the absence of parasitism. However, under parasitism, the relationship of the competitive abilities between *Coix* and host plants was non-significant; additionally, parasitism increased the competitive ability of host plants, but had no effect on the competitive ability of *Coix* (Figure 1a; Table 1). We hypothesize that parasitism may increase the competitive tolerance of host plants to *Coix*, because the competitive suppression of *Coix* by host plants was not influenced by parasitism. However, how parasitism affected the competitive tolerance of host plants merits further study.

In the present study, the competitive abilities of three invasive and native plant species pairs belonging to three families were compared. We found that the competitive ability of two invasive species was lower than their paired native plants in the same family (*Solidago canadensis* vs. *I. polycephala*; *A. philoxeroides* vs. *A. bidentata*), while that of invasive *L. virginicum* was similar to native *Cardamine hirsuta* (Figure S1). When grown without parasitism, the competitive ability of invasive plants in our study was lower than that of native plants. This result was inconsistent with previous studies, that invasive plants have higher competitive abilities than native plants. For example, in the absence of a parasite, invasive plants *S. canadensis* and *A. philoxeroides* are strong competitors and can gradually form monocultures in their invaded ranges [38,39]. The invasive species *L. virginicum* grew larger and produced a higher biomass relative to competitors than the native species did [40]. However, currently, there are increasing studies finding that not all invasive plants were more competitive than native plants [5,41]. For example, Bossdorf et al. [42] found that performance was similar for *Alliaria petiolata* plants from native and invasive populations in the absence of competition, whereas native plants outperformed their invasive conspecifics when competing against each other. We speculated that the higher competitive abilities of native plants in our study may be owing to the identity of competitors [15,41,43]. For example, by quantifying 48 pairs of native and alien plants, Zhang et al. [41] found that common alien plants are more competitive than rare natives but not common natives.

Meanwhile, we found that parasitism increased the competitive ability of invasive plants but had no significant effect on the competitive ability of native plants. This may be owing to *C. grovonii* causing less damage to invasive plants than native plants when under competition. A previous study showed that parasitism may cause more damage to invasive plants than native plants, as the invasive plants have higher resource use efficiency that could support more vigorous growth of parasitic plants [24]. However, several studies showed that invasive plants generally had higher resource use efficiency than native plants [23,44], which can depend on the identity of host plants and the environment [41,45]. More invasive and native plants should be examined in further research when testing the effect of parasitism on competitive ability. Another possible explanation for the present findings may be the invasive status of *C. grovonii* in China. *Cuscuta gronovii*, which is native to North America, was introduced to Beijing, China, in 2010 [27] and is now widely distributed throughout China. The potential co-evolution of *C. gronovii* and its invasive hosts might cause more damage via parasitism to invasive hosts than native hosts.

In this study, we finished the experiment after one-month parasitism, as we observed that *C. grovonii* had caused serious damage on host plants. The competition between host plants and competitors may be changed with time and with the developments of *C. grovonii*. Therefore, we think that studies with different parasitism levels and competition duration are needed in future studies.

## 5. Conclusions

Contrary to our hypotheses, we found that parasitic plants caused less damage to invasive plants than native plants and increased the competitive ability of invasive plants, but not native plants. Our results indicate that parasitic plants can dissimilarly affect the growth of invasive and native plants and may, thereby, alter the competitive dominance among invasive plants and native species. Our results provide an example of how parasitic plants change the structure and function of plant communities, especially invaded communities. Further studies focusing on more invasive and native plant pairs are merited.

## Figures and Tables

**Table 1:** The effects of host invasive status (PS), species (S), and *Cuscuta grovonii* parasitism (CG) on the relative competition intensity (RCI) of host plant and *Coix lacryma-jobi*. RCI was calculated as RCI = (Biomass_alone_ − Biomass_competition_)/Biomass_alone_. *p*-values of statistical significance below 0.05 are indicated in bold.

	Factors	d.f.	*F* Value	*p* Value
RCI of hosts	PS	1, 4	1.983	0.232
	S	4, 60	1.607	0.184
	CG	1, 60	4.185	**0.045**
	PS × CG	1, 60	11.421	**0.001**
RCI of *Coix*	PS	1, 4	8.064	**0.046**
	S	4, 58	1.544	0.202
	CG	1, 58	1.325	0.254
	PS × CG	1, 58	0.153	0.697

**Table 2:** The effects of plant status (PS), species (S) and competition status (C) on the deleterious effect (DE) of *Cuscuta grovonii* on host plants. DE was calculated as (Biomass_parasitized_-Biomass_unparasitized_)/Biomass_unparasitized_. *p* values of statistical significance below 0.05 are indicated in bold.

	Factors	d.f.	*F* Value	*p* Value
DE	PS	1, 4	2.051	0.225
	S	4, 60	2.315	0.068
	C	1, 60	7.058	**0.01**
	PS × C	1, 60	4.19	**0.045**

**Table 3:** The effects of host invasive status (PS), species (S), and competition (C) on the biomass of *Cuscuta grovonii* and on the ratio of *C. grovonii* biomass to host biomass. *p*-values of statistical significance below 0.05 are indicated in bold.

	Factors	d.f.	*F* Value	*p* Value
*C. grovonii* biomass	PS	1, 4	1.01	0.371
	S	4, 44	8.984	**<0.001**
	C	1, 44	0.885	0.352
	PS × C	1, 44	0.418	0.521
Ratio of *C. grovonii* biomass	PS	1, 4	2.8	0.169
	S	4, 44	7.528	**<0.001**
	C	1, 44	1.99	0.165
	PS × C	1, 44	1.569	0.217

## Data Availability

The datasets generated during and/or analysed during the current study are available from the corresponding author upon reasonable request.

## Supplementary Materials

The following supporting information can be downloaded at: https://www.mdpi.com/article/10.3390/life12111800/s1, Figure S1: The relative competitive index (RCI) of the three family-matched pairs of invasive and native plants species in the presence or absence of *Cuscuta grovonii* parasitism. P−, without parasitism; P+, with parasitism. * indicates the RCI index of native and invasive plants belonging to the same family were significantly different (*p* < 0.05); Figure S2: The ratio of *Cuscuta grovonii* biomass to the biomass of host plants when parasitized by *C. grovonii*. LV, *Lepidium virginicum*; AP, *Alternanthera philoxeroides*; SC, *Solidago canadensis*; CH, *Cardamine hirsuta*; AB, *Achyranthes bidentata*; IP, *Ixeris polycephala*. Different lowercase letters indicate significant differences between different host plants
