# Supplementary material for: Effects of Parasitism on the Competitive Ability of Invasive and Native Species
Source: Life (Basel). 2022 Nov 6;12(11):1800. doi: 10.3390/life12111800 (PMC9695417; doi:10.3390/life12111800)
Supplement: Supplementary file 1 [file life-12-01800-s001.zip › life-2004604-supplementary.pdf]

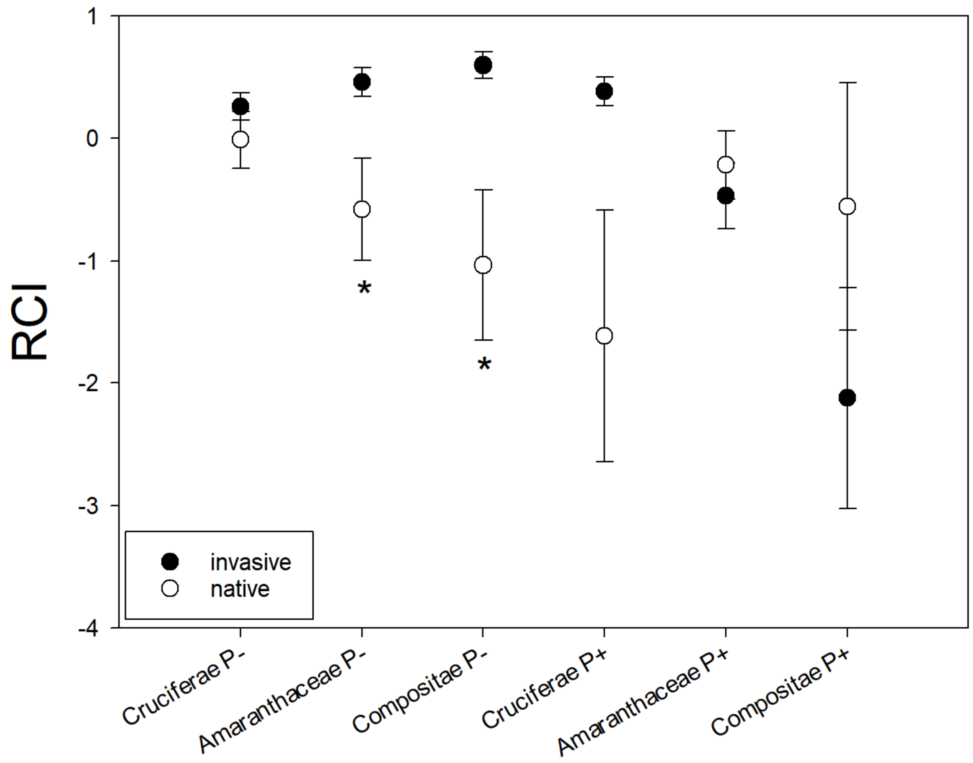

Figure S1. The relative competitive index (RCI) of the three family-matched pairs of invasive and native plants species in the presence or absence of *Cuscuta grovonii* parasitism. P-, without parasitism; P+, with parasitism. * indicates the RCI index of native and invasive plants belonging to the same family were significantly different ($P < 0.05$).

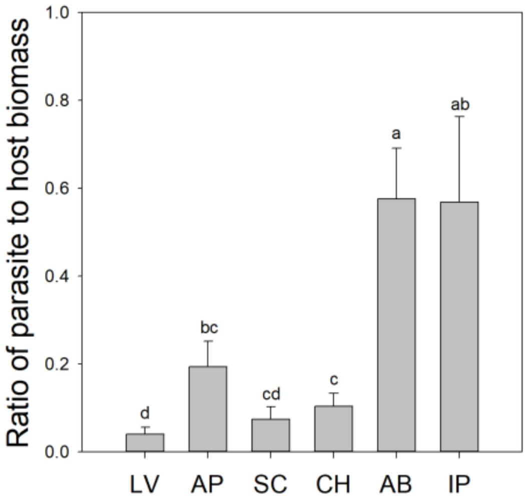

Figure S2. The ratio of *Cuscuta grovonii* biomass to the biomass of host plants when parasitized by *C. grovonii*. LV, *Lepidium virginicum*; AP, *Alternanthera philoxeroides*; SC, *Solidago canadensis*; CH, *Cardamine hirsuta*; AB, *Achyranthes bidentata*; IP, *Ixeris polycephala*. Different lowercase letters indicate significant differences between different host plants.
